# The correlation between transcription factor 7-like 2 gene polymorphisms and susceptibility of gestational diabetes mellitus in the population of central China: A case–control study

**DOI:** 10.3389/fendo.2022.916590

**Published:** 2022-07-29

**Authors:** Pei Zhang, Mengyao Deng, Wei Li, Qiong Dai, Hua He, Wenpei Zheng, Lu She, Bing Xiang, Jing Zeng, Feng Zhou, Yan Guo, Mei Yang

**Affiliations:** ^1^ School of Public Health, Wuhan University of Science and Technology, Wuhan, China; ^2^ Department of Clinical, Bijie Medical College, Bijie, China; ^3^ Maternal and Child Health Hospital of Hubei Province, Tongji Medical College, Huazhong University of Science and Technology, Wuhan, China; ^4^ Department of Chronic Disease, Wuhan Centers for Disease Prevention and Control, Wuhan, China

**Keywords:** genetics, transcription factor 7-like 2, gestational diabetes mellitus, polymorphism, susceptibility

## Abstract

**Objective:**

To investigate the correlation between transcription factor 7-like 2 (TCF7L2) gene polymorphisms and gestational diabetes mellitus (GDM) risk in the central Chinese population.

**Methods:**

This case–control study examined the association of seven *TCF7L2* gene single-nucleotide polymorphisms (SNPs) (rs11196218, rs4506565, rs7895340, rs7901695, rs11196205, rs12243326, and rs290487) with GDM risk in the central Chinese population (843 GDM and 877 controls). The clinical information and blood samples were collected by trained interviewers and nurses. Genotyping of SNPs was conducted on the Sequenom MassARRAY platform. Statistical analyses including *t*-test, ANOVA, chi-square test, Fisher’s exact test, and logistic regression were performed.

**Results:**

Differences in age, pre-pregnant body mass index (BMI), and family history of type 2 diabetes mellitus (T2DM) between the case and control groups were significant (*p* < 0.05). Compared with the wild-type genotype, pregnant women with genotypes of rs4506565-AT (*OR* = 1.89, 95%*CI*: 1.18–3.02), rs7895340 GA (*OR* = 1.93, 95%*CI*: 1.06–3.54), rs7901695-TC (*OR* = 1.79, 95%*CI*: 1.11–2.88), and rs11196205-GC (*OR* = 2.15, 95%*CI*: 1.16–3.98) had a significantly higher risk of GDM, adjusted by age, pre-pregnant BMI, and family history of T2DM. Functional annotation showed that all these four SNPs fell in the functional elements of human pancreatic islets. Further cumulative effects analysis concluded that when participants carried all these four risk genotypes, the risk of GDM was 3.51 times (*OR* = 3.51, 95%*CI*: 1.38–8.90) than that of those without any risk genotypes.

**Conclusions:**

The findings of this study suggested that rs4506565, rs7895340, rs7901695, and rs11196205 were the genetic susceptibility SNPs of GDM in the central Chinese population. Further studies are needed to validate our findings and clarify the underlying mechanisms.

## Introduction

Gestational diabetes mellitus (GDM) is one of the most common complications during pregnancy, showing different degrees of abnormal glucose tolerance. The prevalence of GDM in the Asian population is higher than 20% (1). The prevalence of GDM in the Chinese population has been increasing in recent years. GDM is associated with a variety of adverse outcomes. During the perinatal period, it could increase the risk of preeclampsia, shoulder dystocia, neonatal hypoglycemia, macrosomia, premature delivery, and so on (2). In the long term, the risk of type 2 diabetes mellitus (T2DM) in GDM mothers and their offspring is significantly increased (3, 4).

Transcription factor 7-like 2 (TCF7L2) protein is involved in the stimulation of *β*-cell proliferation and glucagon-like peptide-1 (GLP-1) production. By regulating the Wnt signal pathway, it can stimulate insulin secretion, reduce insulin resistance, and play an important role in maintaining blood glucose homeostasis (5, 6). The common variations in *TCF7L2* gene have been identified as strong predictors of T2DM risk (7–10). In the North Indian population, the researchers concluded that rs7901695 heterozygosity and mutant genotype were associated with an increased risk of T2DM (11). A meta-analysis showed that the rs7903146 T allele was associated with T2DM in the Chinese Han population (12).

Given the consensus that the pathogenesis of GDM is similar to that of T2DM, it is reasonable to hypothesize that the genetic variations associated with β-cell dysfunction and insulin resistance may also contribute to the occurrence of GDM. In recent years, it has aroused researchers’ wide concern about the correlation between TCF7L2 polymorphisms and GDM (5, 6, 13, 14). The results of a meta-analysis confirmed that the rs7903146, rs12255372, and rs7901695 were the most powerful to assess the relationship between TCF7L2 variants and GDM risk, but the contribution of these single-nucleotide polymorphisms (SNPs) to GDM risk varied among different ethnic groups (15). Until now, the role of *TCF7L2* gene polymorphisms in the Chinese population is still unclear.

In order to explore the association between *TCF7L2* gene polymorphisms and GDM susceptibility in Wuhan, central China, we investigated seven SNPs (rs11196218, rs4506565, rs7895340, rs7901695, rs11196205, rs12243326, and rs290487), which have been suggested to be correlated with T2DM but were rarely studied or without consistent conclusions in GDM.

## Materials and methods

### Participants

A total of 1,820 pregnant women (890 GDM and 930 controls) were included in our study. All subjects received prenatal examinations at the Obstetrics and Gynecology Clinic of Maternal and Child Hospital of Hubei Province from January 15, 2018, to March 31, 2019. GDM patients were consecutively recruited, and gestational week-matched controls were randomly selected at the same outpatient during the same time. All participants were enrolled during 24~30 gestational weeks right after the 75-g oral glucose tolerance test (OGTT). GDM was diagnosed according to the criteria of the International Association of Diabetes and Pregnancy Study Groups: fast plasma glucose (FPG) ≥5.1 mmol/L (92 mg/dl), or 1-h result of OGTT (OGTT-1h) ≥10.0 mmol/L (180 mg/dl), or 2-h result of OGTT (OGTT-2h) ≥8.5 mmol/L (153 mg/dl). Pregnant women with age <18, ethnic minorities (41 cases), pre-gestational diabetes (14 cases), multiple pregnancies (18 cases), pregnancy complicated with endocrine diseases such as hypertension (20 cases), any other diseases that could influence glucose regulation (7 cases), and reluctance to participate in this study were excluded. Finally, a total of 1,720 pregnant women were recruited for our study, including 843 GDM and 877 controls. All participants were unrelated Han Chinese and lived in Wuhan, Hubei province.

Informed consent was issued and signed by all participants. The study was conducted in accordance with the Declaration of Helsinki, and the protocol was approved by the Ethics Committee of Wuhan University of Science and Technology (2017002).

### Data collection

At recruitment, trained interviewers used a standard questionnaire to obtain information from all participants, including age, parity, family history of T2DM, current pregnancy status, and other medical problems. Height and pre-gestational weight were obtained through medical records, and pre-gestational body mass index (BMI) was calculated by dividing weight (kg) by height (m) squared.

### Genotyping of single-nucleotide polymorphisms

According to literature studies, seven candidate SNPs (rs11196218, rs4506565, rs7895340, rs7901695, rs11196205, rs12243326, and rs290487) of *TCF7L2* gene that might be associated with GDM risk were selected. At recruitment, 2-ml blood samples from pregnant women were collected after fasting for 8~12 h by skilled nurses. The samples were immediately placed on ice, separated into plasma and cells within 30 min, then divided into equal parts, and stored at −80°C until analysis. Relax Gene Blood DNA System DP348 (Tiangen, China) was used to isolate genomic DNA from 0.5 ml of blood cells. Sequenom MassARRAY platform (Sequenom Inc., San Diego, CA, USA) was applied to genotyping candidate SNPs. For quality control, 5% of duplicate samples were independently reanalyzed in a blinded manner. The call rates of rs11196218, rs4506565, rs7895340, rs7901695, rs11196205, rs12243326, and rs290487 were respectively 98.5%, 99.5%, 99%, 99%, 98.5%, 99%, and 97.5%, which were higher than the quality control standard of 95%.

### *In silico* analysis

To further elucidate the function of the significant SNPs in the pathogenesis of GDM, the Roadmap Epigenomics Project database was used to explore whether the SNPs were located in functional elements.

### Statistical analysis

Normality of distribution for continuous variables was tested by the Kolmogorov–Smirnov test. Then the data conforming to the normal distribution was described by mean ± standard deviation (SD), and the unpaired Student’s t-test or analysis of variance (ANOVA) was used to compare the differences among groups. For qualitative data, the chi-square test or Fisher’s exact test was performed. In addition, the Hardy–Weinberg equilibrium (HWE) was also tested by the chi-square test. The HWE was a principle stating that the participants were representative of the population. The relationship between SNP genotypes and GDM risks was analyzed by multiple logistic regression. All tests were two-sided, and *p* < 0.05 was considered statistically significant. Analyses were performed by using SPSS Software, Version 25.0 for Windows (SPSS Inc., Chicago, IL, USA).

## Results

### Subject characteristics

The basic characteristics of the participants included in the study are shown in **Table 1**. Compared with the control group, GDM patients had higher age, pre-pregnant BMI, FPG, OGTT-1h, and OGTT-2h (*p* < 0.05). Meanwhile, a higher proportion of GDM patients had a family history of T2DM than the control group (30.04% vs. 11.81%, *p* < 0.001).

**Table 1:** The basic characteristics of the participants.

	GDM (N = 843)	Control (N = 877)	*t*/*χ* ^2^	*p*
Age (years)	30.96 ± 4.55	28.81 ± 4.17	10.220	**<0.001**
Pre-pregnant BMI (kg/m^2^)	22.19 ± 3.69	20.64 ± 2.75	8.879	**<0.001**
Gravidity (n/%)			6.165	**0.046**
1	291 (35.44)	345 (52.19)		
2	242 (29.48)	260 (30.20)		
≥3	288 (35.08)	256 (17.61)		
Parity (n/%)			3.412	0.065
Primipara	493 (58.62)	535 (61.14)		
Multipara	348 (41.38)	340 (38.86)		
Family history of T2DM (n/%)			85.913	**<0.001**
No	587 (69.96)	762 (88.19)		
Yes	252 (30.04)	102 (11.81)		
FPG (mmol/L)	5.06 ± 0.87	4.34 ± 0.31	17.654	**<0.001**
OGTT-1h (mmol/L)	10.43 ± 1.71	7.36 ± 1.33	36.258	**<0.001**
OGTT-2h (mmol/L)	9.13 ± 1.75	6.50 ± 0.96	30.542	**<0.001**

Data in bold indicate p < 0.05.

FPG, fasting blood glucose; OGTT-1h, 1-h OGTT blood glucose level; OGTT-2h, 2-h OGTT blood glucose level; OGTT, oral glucose tolerance test; GDM, gestational diabetes mellitus; BMI, body mass index; T2DM, type 2 diabetes mellitus.

### Correlations of candidate single-nucleotide polymorphisms with gestational diabetes mellitus risk and glycemia levels

As shown in **Table 2**, the distributions of SNPs in the control group were all in HWE (*p_HWE_* > 0.05, all). The distributions of genotypes were significantly different between GDM and control groups for rs4506565, rs7901695, rs11196205, and rs12243326. However, after adjustment for age, pre-pregnant BMI, and family history of T2DM, pregnant women with genotypes of rs4506565-AT (*OR* = 1.89, 95%*CI*: 1.18–3.02), rs7895340 GA (*OR* = 1.93, 95%*CI*: 1.06–3.54), rs7901695-TC (*OR* = 1.79, 95%*CI*: 1.11–2.88), and rs11196205-GC (*OR* = 2.15, 95%*CI*: 1.16–3.98) had a significantly higher risk of GDM.

**Table 2:** Association between *TCF7L2* gene SNPs and GDM risk.

SNPs	Genotypes	GDM/control	HWE (*X* ^2^)	*p_HWE_*	Model 1		Model 2	
					*OR* (95%CI)	*p*	*OR* (95%CI)	*p*
rs11196218	GG	416/455	0.35	0.84	Ref.		Ref.	
	GA	330/345			1.05 (0.86, 1.28)	0.660	0.99 (0.78, 1.25)	0.902
	AA	71/59			1.32 (0.91, 1.91)	0.146	1.42 (0.90, 2, 24)	0.128
rs4506565	AA	745/815	0.11	0.95	Ref.		Ref.	
	AT	74/48			**1.68 (1.15, 2.45)**	**0.007**	**1.89 (1.18, 3.02)**	**0.008**
	TT	2/1			2.18 (0.20, 24.08)	0.525	1.08 (0.07, 15.74)	0.954
rs7895340	GG	772/829	0.29	0.87	Ref.		Ref.	
	GA	45/31			1.56 (0.98, 2.49)	0.063	**1.93 (1.06, 3.54)**	**0.033**
rs7901695	TT	745/813	0.17	0.92	Ref.		Ref.	
	TC	70/46			**1.66 (1.13, 2.44)**	**0.010**	**1.79 (1.11, 2.88)**	**0.018**
	CC	72/1			2.18 (0.20, 24.12)	0.524	1.08 (0.07, 15.60)	0.957
rs11196205	GG	768/828	0.25	0.88	Ref.		Ref.	
	GC	45/29			**1.67 (1.04, 2.70)**	**0.034**	**2.15 (1.16, 3.98)**	**0.015**
rs12243326	TT	805/852	0.01	0.99	Ref.		Ref.	
	TC	15/6			**2.65 (1.02, 6.85)**	**0.045**	2.36 (0.63, 8.81)	0.203
rs290487	TT	282/306	0.01	0.99	Ref.		Ref.	
	TC	401/406			1.07 (0.87, 1.33)	0.523	1.09 (0.85, 1.41)	0.487
	CC	124/137			0.98 (0.73, 1.32)	0.904	0.93 (0.66, 1.32)	0.690

Data in bold indicate p < 0.05.

HWE, Hardy–Weinberg equilibrium test; Model 1, unadjusted; Model 2, adjusted by age, pre-pregnant BMI, and family history of T2DM; SNPs, single-nucleotide polymorphisms; GDM, gestational diabetes mellitus; BMI, body mass index; T2DM, type 2 diabetes mellitus.

**Figure 1:**
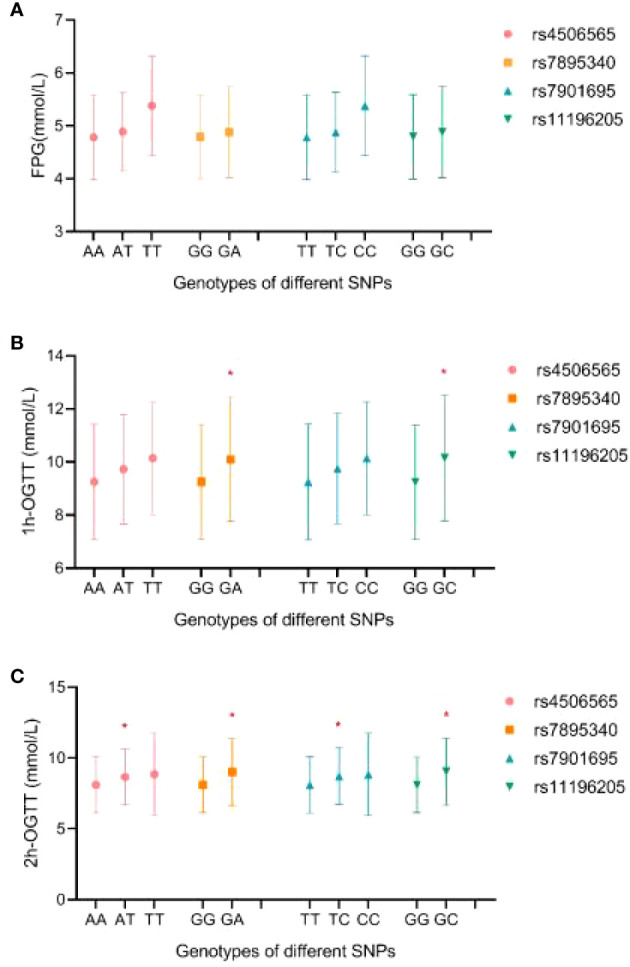
The distribution of FPG **(A)**, OGTT-1h **(B)**, and OGTT-2h **(C)** levels by different genotypes. FPG, fast plasma glucose; OGTT-1h, 1-h result of oral glucose tolerance test; OGTT-2h, 2-h result of oral glucose tolerance test. *Compared with homozygous of major allele, p < 0.05.

The levels of FPG, OGTT-1h, and OGTT-2h by different genotypes are shown in **Figure 1**. There were no significant differences in FPG levels among genotypes for each SNP (*p* > 0.05). Pregnant women with rs7895340 GA genotype and rs11196205-GC genotype had significantly higher OGTT-1h levels than wild type (*p* = 0.004 and 0.002, respectively). For OGTT-2h level, carriers with heterozygous genotype were significantly higher than carriers with homozygote of major alleles in all four SNPs (*p* = 0.043, 0.001, 0.022, and 0.001).

### Functional annotation

**Figure 2:**
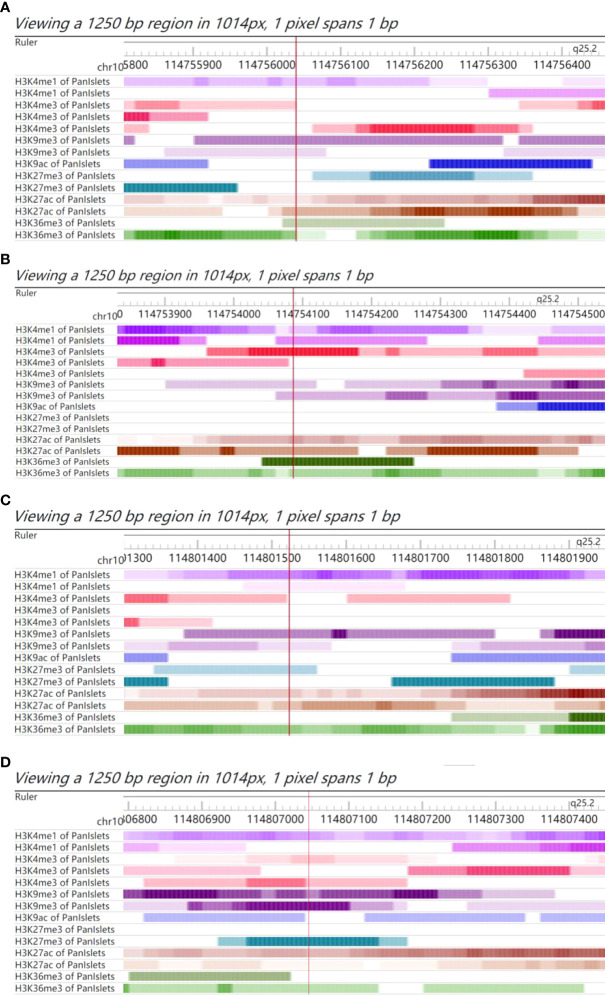
Chromatin features of rs4506565, rs7901695, rs7895340, and rs11196205. Functional annotation in proximity to rs4506565 **(A)**, rs7901695 **(B)**, rs7895340 **(C)**, and rs11196205 **(D)** location in pancreatic islets from Roadmap data. ChIP-seq tracks (H3K4me1, H3K27ac, H3K4me3, H3K9me3, and H3K27me3) for histone marks are present. The red vertical lines guide the position of rs4506565, rs7901695, rs7895340, and rs11196205. The colored stripes represent the density signal.

The functional annotations of rs4506565, rs7895340, rs7901695, and rs11196205 were explored using the Roadmap database. We observed that these four SNPs all fell in the functional regulatory elements of human pancreatic islets, including several histone modification markers such as histone 3 lysine 4 mono-methylation (H3K4me1), histone 3 lysine 27 acetylation (H3K27ac), histone 3 lysine 4 tri-methylation (H3K4me3), histone 3 lysine 9 tri-methylation (H3K9me3), and histone 3 lysine 27 tri-methylation (H3K27me3). As presented in **Figure 2**, rs4506565 is located in the H3K4me1, H3K4me3, H3K9me3, H3K27ac, and H3K36me3. rs7901695 is located in the H3K4me1, H3K4me3, H3K9me3, H3K27ac, and H3K36me3. rs7895340 is located in the H3K4me1, H3K9me3, H3K27me3, H3K27ac, and H3K36me3. rs11196205 is located in the H3K4me1, H3K4me3, H3K9me3, H3K27me3, H3K27ac, and H3K36me3.

### Cumulative effects of risk genotypes in gestational diabetes mellitus

The cumulative effect analysis indicated that the risk of GDM was 2.51-fold higher in pregnant women who carried four risk genotypes than that of those without any risk genotypes (*OR* = 3.51, 95%*CI*: 1.38–8.90), as shown in **Table 3**.

**Table 3:** Cumulative effects of risk genotypes in GDM.

The number of risk genotypes	GDM/control	Model 1		Model 2	
		*OR* (95%*CI*)	*p*	*OR* (95%*CI*)	*p*
0	752/807	1.00 (Ref)		1.00 (Ref)	
1	4/3	1.43 (0.32–6.41)	0.640	1.18 (0.23–5.97)	0.840
2	56/57	1.05 (0.72–1.55)	0.786	1.40 (0.87–2.26)	0.172
3	2/1	2.15 (0.19–23.72)	0.533	0.47 (0.04–6.15)	0.564
4	29/9	**3.46 (1.63–7.35)**	**0.001**	**3.51 (1.38–8.90)**	**0.008**

Data in bold indicate p < 0.05.

Model 1, unadjusted; Model 2, adjusted by age, pre-pregnant BMI, and family history of T2DM; GDM, gestational diabetes mellitus; BMI, body mass index; T2DM, type 2 diabetes mellitus.

## Discussion

In the present study, we investigated the correlation between *TCF7L2* gene SNPs (rs11196218, rs4506565, rs7895340, rs7901695, rs11196205, rs12243326, rs290487) and the risk of GDM in Wuhan, a central area of China. The results showed that mutations in rs4506565, rs7895340, rs7901695, and rs11196205 might be related to a higher risk of GDM in the central Chinese population. The mutations in these four SNPs might also be associated with higher OGTT-1h or OGTT-2h blood glucose levels. Moreover, when participants carried all these four risk genotypes, the risk of GDM was 3.51 times that of those without any risk genotypes.

Previous studies suggest that mutations in TCF7L2 can reduce insulin secretion, disrupt the homeostasis of blood glucose, and significantly increase the risk of T2DM (16). The seven SNPs we studied have been confirmed to be associated with T2DM risk in different populations (17–22). However, the correlations between the seven SNPs and the risk of GDM are still inconsistent. Social-demographic characteristics, diagnosis standard, genetic background, sample size, and so on might be the confounding factors that affected the consistency of the results. It is necessary to further study the relationship between TCF7L2 polymorphisms and GDM risk in the Chinese population.

As far as we know, this was the first study to explore the correlation between rs7895340 polymorphism and GDM risk in Chinese, and the result was significantly positive. Our study reported that rs4506565 was associated with an increased risk of GDM in the central Chinese population, which was consistent with the results from the Scandinavian and Mexican populations (13, 23). Our results also concluded that rs11196205 was significantly associated with GDM risk, but in the Northern Chinese population, this association was negative (24). In addition, compared with the TT genotype, the rs7901695-TC genotype was found to be associated with higher GDM risk in our Chinese population, similar to the results of the Swedish population (14). However, in American Caucasians, the correlation between the two was inverse, which showed the T allele of rs7901695 might increase the risk of GDM by 0.98 times compared with the C allele (25). The other two studies in a population of Brazil and Southern Poland did not find a significant correlation between rs7901695 polymorphism and GDM risk (5, 6).

In the study of Amira Turki et al., they found that when participants carried multiple risk alleles, the T2DM risks would be higher or lower compared with single-site variation (19). In our study, 10.32% of GDM patients had two or more risk genotypes at the same time. There was a significant cumulative effect of risk genotypes on GDM.

*In silico* analysis concluded that the four positive SNPs are all located in the functional elements of human pancreatic islets, which hinted that the mutation in these SNPs might affect insulin secretion. The ability of insulin secretion was very important for the regulation of postprandial blood glucose levels. Similarly, our results concluded that participants who carried genotypes of rs4506565-AT, rs7901695-TC, rs7895340-GA, and rs11196205-GC had significantly higher OGTT-1h or OGTT-2h levels, but not FPG levels. Glucose homeostasis is a complex trait that could be influenced by both genetic and environmental factors. The potential mechanisms of TCF7L2 polymorphism in blood glucose homeostasis still need further study.

While a relatively systematic study was used to evaluate the association between seven TCF7L2 candidate SNPs and GDM risk in the Chinese population, which was rarely studied, our study also had some limitations. Firstly, although we had a relatively large sample size, the minor allele frequencies of some candidate SNPs were small, resulting in the low power of the results. Secondly, the level of TCF7L2 and insulin were not measured. Thirdly, the information on environment and lifestyle factors was not analyzed, which had been reported to be important determinants of GDM development. Finally, although functional annotation of candidate SNPs was conducted based on the public database, the underlying mechanisms need to be revealed by in-depth functional experiments.

## Conclusions

In conclusion, the results of this study indicated that the *TCF7L2* gene SNPs rs4506565, rs7895340, rs7901695, and rs11196205 were genetic susceptibility SNPs to GDM in the central Chinese population, which might provide a new perspective for the prevention of GDM.

## Data availability statement

The datasets used and/or analyzed during the current study are available from the corresponding authors on reasonable request. Requests to access these datasets should be directed to Mei Yang, 644593079@qq.com.

## Ethics statement

The studies involving human participants were reviewed and approved by the Ethics Committee of Wuhan University of Science and Technology (2017002). The patients/participants provided their written informed consent to participate in this study.

## Author contributions

PZ, MD, and WL contributed to the study design, analysis, and interpretation of data and drafted the manuscript. QD, HH, WZ, LS, BX, JZ, and FZ performed data collection and interpretation. YG and MY participated in the design and coordination of the study and revised the manuscript. All authors read and approved the final manuscript.

## Acknowledgments

We appreciated all the study participants, hospital workers, research staff, and students who participated in this work. This work was supported by the National Natural Science Fund of China (81703239), Education Department of Hubei Province (B2020006), and Health Commission of Hubei Province (WJ2018H0134, WJ2018H0145).

## Conflict of interest

The authors declare that the research was conducted in the absence of any commercial or financial relationships that could be construed as a potential conflict of interest.

## Publisher’s note

All claims expressed in this article are solely those of the authors and do not necessarily represent those of their affiliated organizations, or those of the publisher, the editors and the reviewers. Any product that may be evaluated in this article, or claim that may be made by its manufacturer, is not guaranteed or endorsed by the publisher.
